# Effects of White and Blue-Red Light on Growth and Metabolism of Basil Grown under Microcosm Conditions

**DOI:** 10.3390/plants12071450

**Published:** 2023-03-25

**Authors:** Luigi d’Aquino, Rosaria Cozzolino, Giovanni Nardone, Gianni Borelli, Emilia Gambale, Maria Sighicelli, Patrizia Menegoni, Giuseppe Carlo Modarelli, Juri Rimauro, Elena Chianese, Giuseppe Nenna, Tommaso Fasolino, Gilda D’Urso, Paola Montoro

**Affiliations:** 1 ENEA, Portici Research Centre, Piazzale E. Fermi 1, Napoli, 80055 Portici, Italy; 2 Institute of Food Science, National Council of Research, Via Roma 64, 83100 Avellino, Italy; 3 FOS S.p.A., Via E. Melen 77, 16152 Genova, Italy; 4 Becar S.r.l. (Beghelli Group), Viale della Pace 1, Monteveglio, 40050 Bologna, Italy; 5 ENEA, Casaccia Research Centre, Via Anguillarese 301, Santa Maria di Galeria, 00060 Roma, Italy; 6 Department of Agricultural Sciences, University of Naples Federico II, Via Università 100, Napoli, 80055 Portici, Italy; 7 Department of Science and Technology, University of Naples Parthenope, Isola C4, Centro Direzionale di Napoli, 80143 Napoli, Italy; 8 Department of Pharmacy, University of Salerno, Via Giovanni Paolo II, Salerno, 84084 Fisciano, Italy

**Keywords:** LED lighting, indoor farming, plant nutrients, plant metabolomics, Chl *α* fluorescence, precision agriculture

## Abstract

Indoor farming of basil (*Ocimum basilicum* L.) under artificial lighting to support year-round produce demand is an area of increasing interest. Literature data indicate that diverse light regimes differently affect downstream metabolic pathways which influence basil growth, development and metabolism. In this study, basil was grown from seedlings to fully developed plants in a microcosm, an innovative device aimed at growing plants indoor as in natural conditions. Specifically, the effects of white (W) and blue-red (BR) light under a photosynthetic photon flux density of 255 μmol m^−2^ s^−1^ on plant growth, photochemistry, soluble nutrient concentration and secondary metabolism were investigated. Plants grew taller (41.8 ± 5.0 vs. 28.4 ± 2.5 cm) and produced greater biomass (150.3 ± 24.2/14.7 ± 2.0 g vs. 116.2 ± 28.3/12.3 ± 2.5 g fresh/dry biomass) under W light compared to BR light. The two lighting conditions differently influenced the soluble nutrient concentration and the translocation rate. No photosynthetic stress was observed under the two lighting regimes, but leaves grown under W light displayed higher levels of maximum quantum yield of PSII and electron transport rate. Sharp differences in metabolic patterns under the two lighting regimes were detected with higher concentrations of phenolic compounds under the BR light.

## 1. Introduction

In recent times, indoor farming, after the initial interest in space colonization by humans [1], has met increasing interest among farmers as a practical and effective approach to support year-round produce demand in urban environments, and also to face the adverse effects of climate change on conventional agricultural systems [2,3,4,5,6,7]. So far, the development of indoor farming technology has been mainly based on progress in artificial lighting and environmental controlling/sensing, which enable farmers to grow plants under totally controlled environments. Plant lighting based on light emitting diodes (LEDs) is probably the most relevant technological progress in this field, thanks to several traits, including smaller lamp sizes, lower heat emissions, greater photonic emission efficiency, quicker reaching the stationary radiant flux, longer life span and easier connection to digital control systems compared to conventional light sources [8,9,10,11,12,13]. LED technology also enables researchers to supply plants with selected wavelengths, allowing more accurate investigation of the effects of unconventional light regimes on yield levels and biomass quality. Scientific literature suggests that, in the future, indoor farmers could modify light regimes to drive plant growth, development and metabolism to better meet consumer needs [14,15,16,17,18,19,20,21,22].

Basil (*Ocimum basilicum* L.; Fam. *Lamiaceae*) has been used as a culinary herb since prehistoric times, and today it is considered a highly valued horticultural crop worldwide. It also holds a prominent position as a medicinal plant, owing to a high content of biologically active compounds, including well-recognized antimicrobial and antioxidant compounds [23,24]. Small dimensions, high growth rate and short cultivation cycle make basil an interesting crop for indoor farming which can support year-round demand for basil as a fresh and dry herb, pesto sauce, extracts and essential oil. The scientific literature has clearly demonstrated that different lighting regimes affect downstream metabolic pathways which influence basil growth, development and metabolism, allowing one to tune basil metabolism by modulating light quality and intensity [14,21,22,23,24,25,26,27,28,29,30,31,32,33,34,35,36,37,38,39,40,41,42,43,44,45,46,47,48,49,50,51,52,53,54,55,56,57,58]. Unravelling the huge amount of experimental literature results is, however, a very challenging task, as they were generated from research approaches which differ in many aspects, including experimental design and set-up, germplasm, plant developmental stages, duration of cultivation and growing methods, in addition to light supply. Therefore, no universally accepted lighting protocols for indoor basil farming are currently available, which is also due to the very few experiments on long-lasting cultivations, as in real crop conditions.

Currently, the approach in indoor farming is widely based on soilless technologies, such as hydroponics, aeroponics and, more recently, aquaponics. In this context, a new concept, known as “microcosm”, aimed at growing plants indoors as in natural conditions has been recently developed (European patent n. 3236741). Briefly, a microcosm device is made up of two chambers, one for housing the epigeal plant part and another for housing the roots. In both chambers, temperature, light and air-flow regimes can be independently set and controlled, enabling the operators to grow roots and aerial parts of the plants under different environmental conditions. Plant roots are grown in deep cylindrical pots with the bottoms replaced by grid funnels to prevent limitations to root deepening. The height of the two chambers allows basil-like plants to reach a shoot height/root depth ratio of about 1.5, permitting natural development of the aerial part. In previous work, basil was grown in a microcosm from seedlings to the flowering stage to evaluate the efficacy of this technology on yield, photochemistry, soluble nutrients concentration and secondary metabolism. The results confirmed that the new growing approach is suitable for indoor basil growing and for long lasting cultivation, as plants presented a biomass yield and quality similar to those achievable in typical open-field or greenhouse crops [33], as previously demonstrated in potato (*Solanum tuberosum* L.) [59].

The subsequent technological evolution of the microcosm enabled researchers to achieve a more accurate regulation of the light supply in the epigeal chamber, to balance wavelength ratios and light intensity, compared to the basic prototype previously tested [33]. Thanks to this improvement, as a contribution to assess the most suitable lighting protocols for indoor basil farming, in the present study, basil plants were grown in parallel in two microcosms. In one device, basil was cultivated under white (W) light, while in the other microcosm plants were raised under blue-red (BR) light. Both W and BR light displayed the same photosynthetic photon flux density (PPFD) of 255 μmol∙m^−2^∙s^−1^. Basil was grown from the seedling stage until the beginning of flowering to simulate cultivations targeted to the production of herbs or pesto ingredient, in which exploitation of all the potential of leaf and stem production is requested by farmers. The effects of the two light spectra were assessed by measuring the biomass yield, as this is the first feature the farmers are interested in, and detecting the soluble nutrient and secondary metabolite contents, as these features affect the global quality of the final product. Since biomass yield and quality are both related to metabolic pathways driven by photosynthesis, the response of the photosynthetic apparatus to the different lighting conditions was also tested.

## 2. Results

### 2.1. Plant Growth and Development

**Figure 1:**
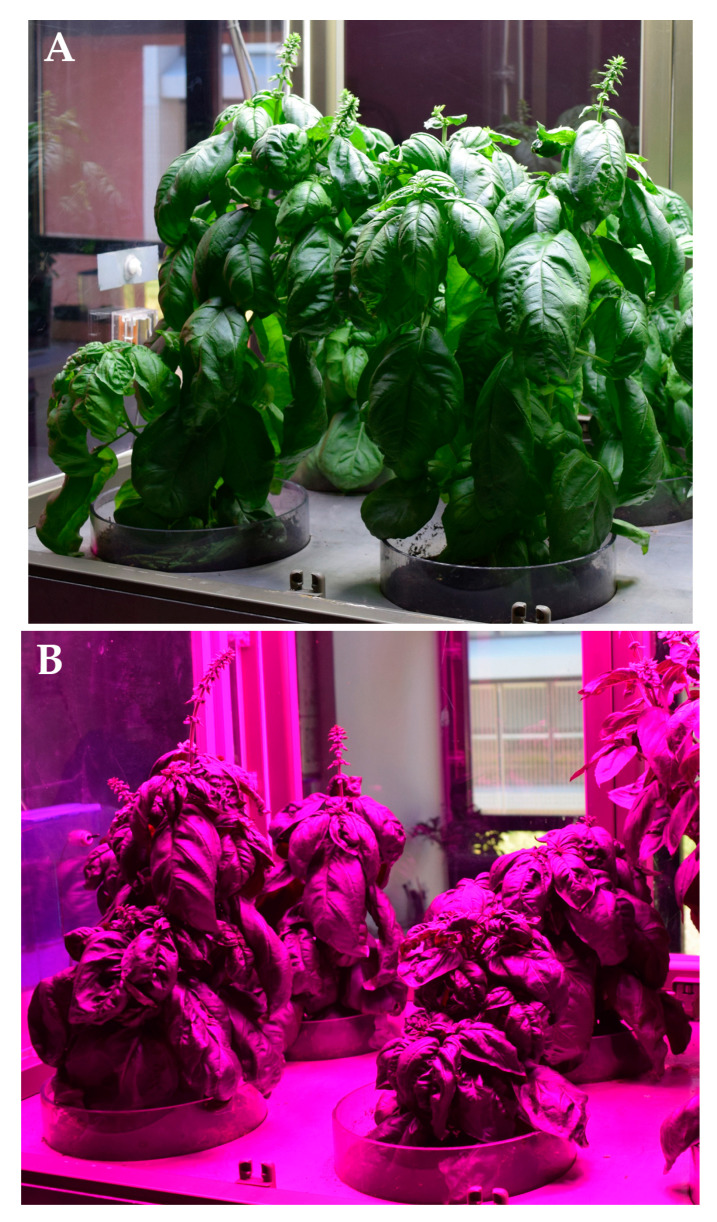
Basil plants in the microcosms under W (**A**) and BR (**B**) light at the harvest stage.

Basil plants grew healthy and vigorously in the two microcosms for the whole cultivation period. They reached their maximum height at the beginning of the flowering stage, i.e., at the emission of the inflorescence axes (Figure 1), which occurred 58 days after the transplant.

**Figure 2:**
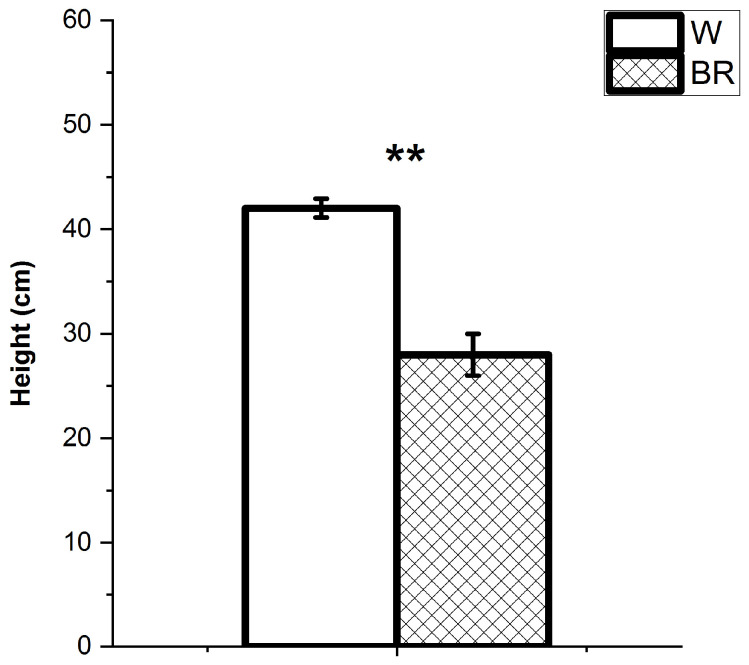
Average height of plants cultivated under W and BR light determined at the harvest stage. Bars represent the mean values and the standard errors (SE) (n = 6 replicates). A significant difference (*p* ≤ 0.01) is indicated as **.

**Figure 3:**
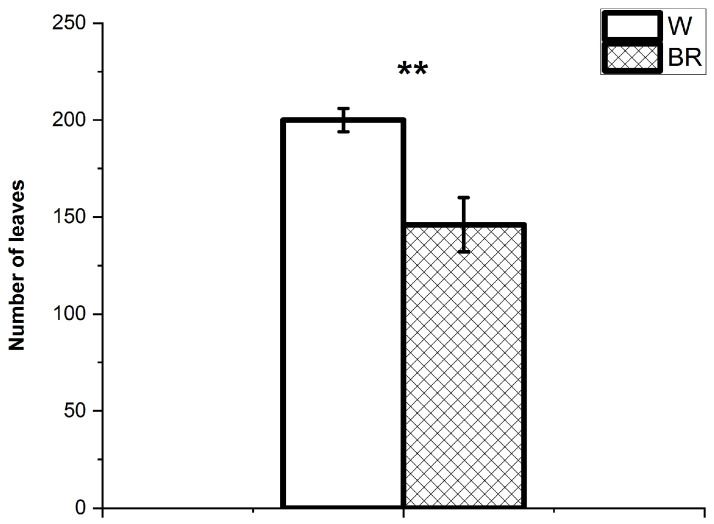
Average number of leaves per plant under W and BR light at the harvest stage. Bars represent the mean values and the standard errors (SE) (n = 6 replicates). A significant difference (*p* ≤ 0.01) is indicated as **.

**Figure 4:**
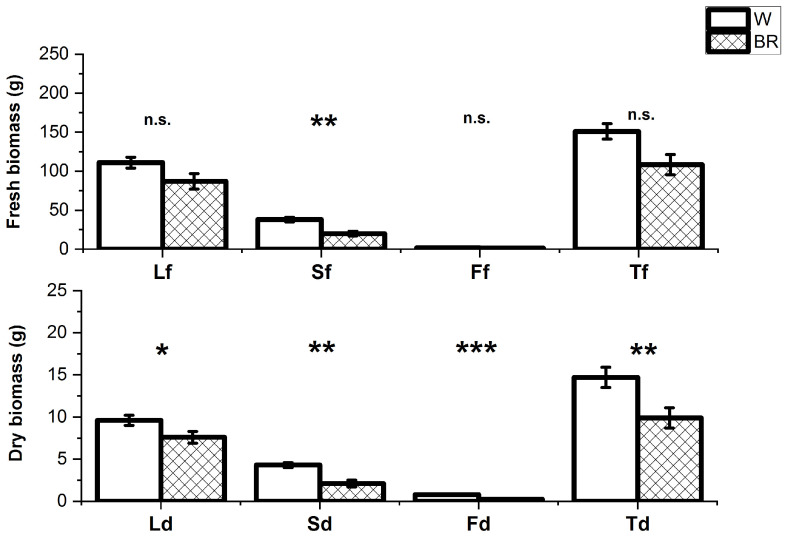
Average fresh (f, upper) and dry (d, lower) weights of leaves (L), stems (S) and flowers (F) and average weights of the total aerial biomasses (T) of plants grown under W and BR light determined at the harvest stage. Bars show the mean values and the SE (n = 6 replicates). Not significant and significant differences at *p* ≤ 0.05, 0.01 or 0.001 are indicated as ns, *, ** and ***, respectively.

The two lighting conditions differently affected the plant height at the harvest stage differently. Specifically, the plants grown under the W light were taller than those under the BR light (average height cm 41.8 ± 5.0 and 28.4 ± 2.5, respectively) (Figure 2). The average number of leaves per plant was also significantly higher in the plants grown under the W light compared to the BR light (200.5 ± 15.4 and 146.4 ± 30.4, respectively) (Figure 3). The average fresh and dry weights of aerial organs are reported in Figure 4. The average total fresh weights of the aerial parts under W and BR light were about 150.3 ± 24.2 g and 116.2 ± 28.3 g, respectively, whereas the corresponding dry weights were about 14.7 ± 2.0 g and 12.3 ± 2.5 g, respectively.

### 2.2. Chlorophyll Fluorescence Analysis

**Figure 5:**
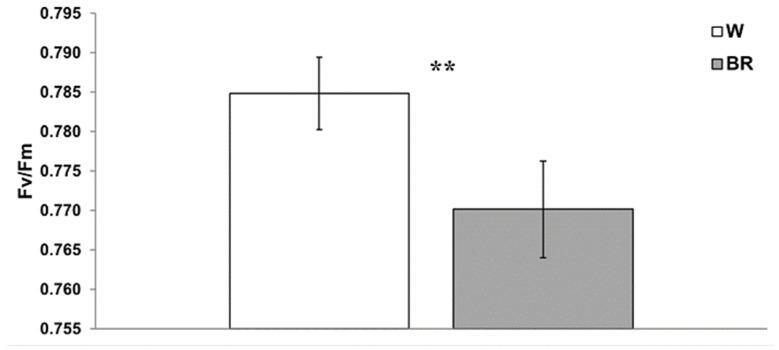
Mean Fv/Fm values ± SE (n = 3) under W and BR light measured 28 days after the transplant. A significant difference (*p* ≤ 0.006) is indicated as **.

**Figure 6:**
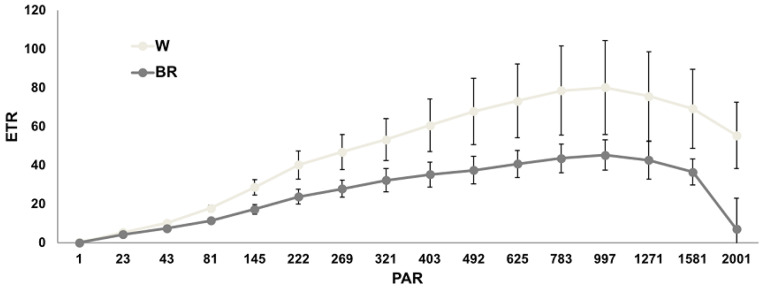
Mean ETR values ± SE (n = 3) measured 28 days after the transplant under W and BR light. No significant differences were detected.

The maximum photochemical efficiency (Fv/Fm) and the electron transport rate (ETR) were measured on middle fully expanded leaves 28 days after transplant, and the results are shown in Figure 5 and Figure 6, respectively.

The Fv/Fm ratio reflects the maximum quantum efficiency of PSII. The recorded mean Fv/Fm values ranged between 0.770 and 0.785, indicating no detectable photosynthetic stress in plants under both lighting regimes. As the light induction curves showed, basil grown under the W light displayed a higher electron transport rate (ETR) as compared to BR, where the maximum ETR values were reached at 997 μmol·m^−2^·s^−1^ of actinic light.

### 2.3. Nutrient Concentration

Table 1 reports the concentrations of the soluble fractions of several main nutrients plus Na^+^ and Cl^−^ ions, determined at the harvest stage in the stems and leaves of plants grown under the two lighting regimes.

All the ionic species considered in this study displayed a similar distribution pattern between leaves and stems under both W and BR light, with higher concentrations in leaves, except for the Na^+^ ions. Total ions were particularly elevated in plants grown under W light, especially in stems. The leaves/stems concentrations ratios ranged from 0.01 (Na^+^) to 1.92 (Mg^2+^) and from 0.01 (Na^+^) to 3.00 (Mg^2+^) under W and BR light, respectively.

### 2.4. Metabolic Profiling

**Figure 7:**
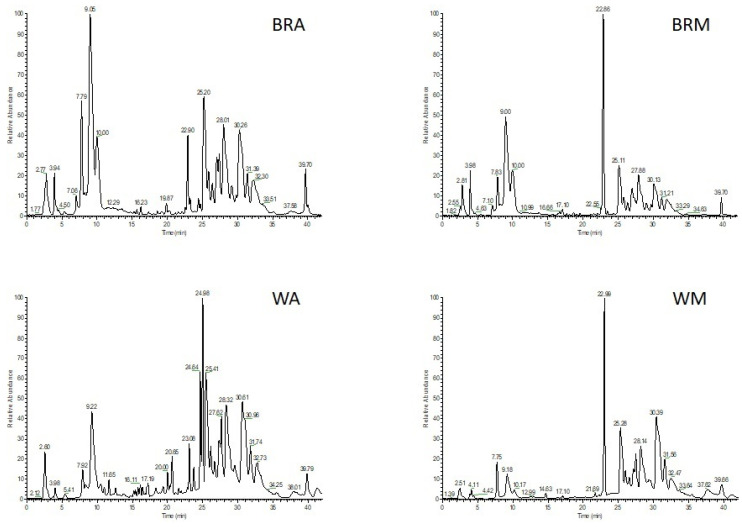
LC-ESI-Orbitrap-MS profiles in negative ionization mode of leaf samples collected at the harvest stage. BRA, apical leaves of plants from microcosm BR; BRM, middle leaves of plants from microcosm BR; WA, apical leaves of plants from microcosm W; WM, middle leaves of plants from microcosm W.

The metabolic patterns of hydroalcoholic extracts achieved from the apical (A) and middle (M) leaves grown under BR and W light obtained by LC-ESI-FT-(Orbitrap)-MS analysis in negative ionization mode are reported in Figure 7.

High-resolution mass spectrometry analysis followed by MS/MS fragmentation experiments allowed detection of 30 main compounds, mainly belonging to flavonoids, phenylpropanoids, organic acids and catechin, in addition to stilbenes and two triterpenic acids (Table 2).

**Figure 8:**
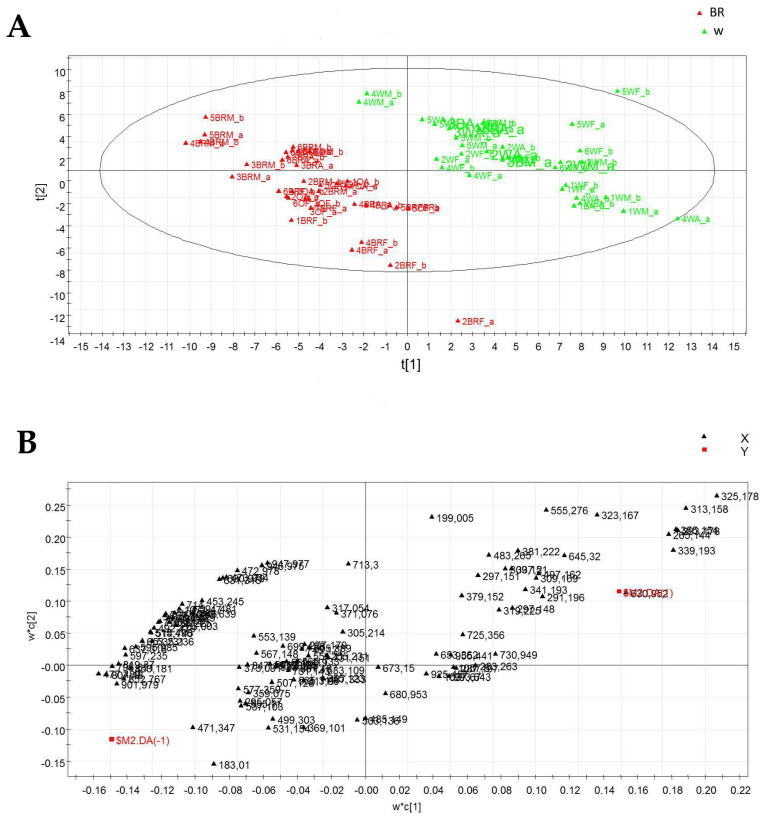
Score scatter plot (**A**) and loading plot (**B**) of the untargeted PLS-DA performed on the LC-ESI/LTQOrbitrap/MS data.

The LC-ESI/LTQOrbitrap/MS data were subjected to multivariate analysis using PLS-DA as a projection method, and the score scatter plot is reported in Figure 8A. The first component expressed 31% of the variance, whereas the second component accounted for 14% of the variance, allowing discrimination of the samples in two main clusters. The metabolites detected in plants grown under the W light were mainly distributed in the right part of the plot, while those observed in basil grown under the BR light were mostly spread in the left part of the plot. Due to the large number of variables, no marker compounds could be selected in the loading plot obtained from the untargeted PLS-DA analysis, as shown in Figure 8B.

**Figure 9:**
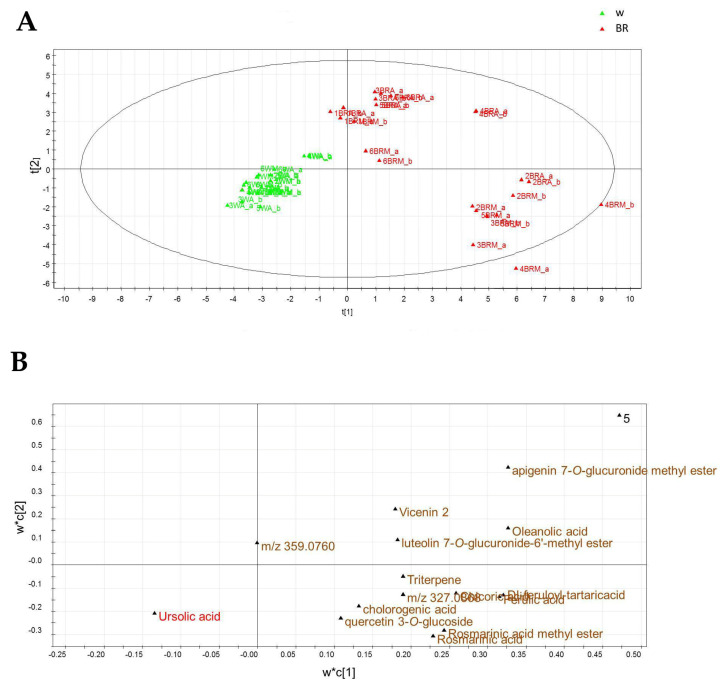
Score scatter plot (**A**) and loading plot (**B**) of the targeted PLS-DA performed on the LC-ESI/LTQOrbitrap/MS data.

To identify marker metabolites useful to discriminate samples from plants grown under the two different lighting regimes, a pseudo-targeted approach was developed by building a new data matrix considering and manually measuring the areas of the peaks of the compounds listed in Table 2. This matrix was then submitted to multivariate analysis by the SIMCA-P+ software using both PCA and PLS-DA. The score scatter plot of the targeted PLS-DA analysis (Figure 9A) allowed to differentiate the samples into two groups, one from the plants grown under the W light (WA and WM), positioned in the left quadrants, and one from the plants grown under the BR light (BRA and BRM), positioned in the right quadrants, confirming the results of the untargeted analysis. An additional separation was obtained between the BRA samples that were positioned in the upper right side of the plot and the BRM samples that were placed in the lower right part. The results from the loading plot analysis (Figure 9B) indicated that the phenolic compounds were more expressed in BRA and BRM samples.

## 3. Discussion

The experimental set-up used in this study was suitable to investigate the effect of different lighting regimes on fully expanded basil plants, which represents the less investigated plant model in the field of plant-light interaction compared to smaller plants and microgreens. An experimental design based on W vs BR light under the same PPFD level was chosen as a first test of the effect of different light spectra on basil yield and quality. Biometric determinations indicated that the average dry biomass of all the aerial organs was higher in plants grown under W light. This finding disagrees with the results reported by other authors, who indicated better growth performance in basil microgreens and in basil grown in small pots under selected wavelengths or blue-red light. Different results can be due to different basil genotypes and different lighting conditions (for example, B:R ratios, PPFD levels, photoperiod, etc.) as well as to different plant developmental stages and canopy widths [26,37,42]. In particular, the greater growth of basil under W light observed in the present study can also be related to the better attitude of the white light to reach the lower and inner parts of the plants compared to the blue and red wavelengths alone [60]. Chlorophyll fluorescence analysis was carried out on middle fully expanded leaves at about half of the expected vegetative growing period (4 weeks after transplant), in order to evaluate the effect of the lighting regimes on photosynthetic systems when plants were still in the active growing period [61]. The recorded Fv/Fm values indicated that neither W nor BR lights caused any photosynthetic stress to the plants, while the W light resulted in elevated electron transport, in line with previous results about the effect of different light spectra on PSII quantum efficiency [21,62,63,64]. The concentrations of the soluble fractions of several main nutrients detected in the plants at the end of the growing period agree with the values already reported in basil by Yang and Kim [65]. The greater amount of the total ions in plants grown under W light, especially in stems, is consistent with the higher biomass production displayed by these plants. The soluble nutrient concentrations were influenced by the lighting conditions, except for Na^+^ in the leaves. Markedly, the plants grown under W light accumulated higher levels of NO_3_^−^, K^+^ and Cl^−^, and lower levels of NH_4_^+^, P, PO_4_^3−^, Mg^2+^, Ca^2+^, S, SO_4_^2−^. The leaf-to-stem concentration ratios were higher under the BR light for all the tested elements, except for S and SO_4_^2−^, thus suggesting an enhancing effect of the BR light on the translocation rate.

The importance of a metabolomics approach to evaluate the plant response to environmental factors is receiving increasing acknowledgement by the scientific literature [57]. Specifically, the metabolomics of fully expanded plants is less investigated compared to smaller plants and microgreens. To investigate the effect of the W and BR light on the secondary metabolism of the fully expanded basil plants, a metabolomics approach based on LC-ESI-Orbitrap-MS combined with a multivariate data analysis was followed. As expected, different metabolic profiles in middle and apical leaves collected from plants grown under W and BR light were recorded. Sharp differences were observed in the metabolite distribution following the multivariate analysis, confirming that the two lighting regimes differently affected the secondary metabolism in basil. In this study, an untargeted metabolomics approach was chosen, as it enables the identification of key changes in metabolic pathways and helps to reveal important and putative novel metabolites or pathways for the implementation of further analyses. Notwithstanding the great progress that has been made in this field over the past decade, plant metabolomics with an untargeted approach still seems to be a valuable approach as it can generate comprehensive information regardless of the high complexity of plant metabolites [66,67,68,69]. Among the compounds responsible for the separation of the samples obtained from plants grown under BR light, several molecules with peculiar biological and pharmacological properties were identified. In particular, antioxidant activity and beneficial effects on spermatogenesis were described for ellagic acid (Table 2, compound **7**) and chlorogenic acid (Table 2, compound **9**) [70], antioxidant, anti-inflammatory, antiviral and immune-stimulating properties were reported for chicoric acid (Table 2, compound **8**) [71], already detected in different basil organs [72] and antiviral, antimicrobial and anti-inflammatory activities were observed for rosmarinic acid (Table 2, compound **13**) [53]. Higher levels of these compounds were detected in plants grown under BR light, thus suggesting that the BR light can promote the production of phenolic compounds associated with interesting biological activities.

## 4. Materials and Methods

### 4.1. Germplasm and Growing Conditions

**Figure 10:**
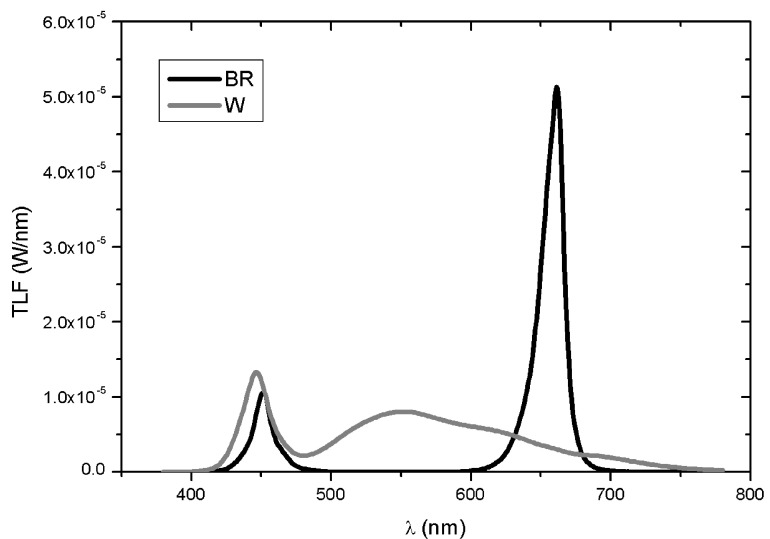
Total luminance flux (TLF, W/nm) at 87 cm from the light sources in the microcosm W (grey line) and BR (black line) in the region λ 350 ÷ 800 nm.

Seedlings of basil type Genovese cv. “Bonsai” (Blumen Vegetable Seeds, Milano, Italy), a rather common Italian variety of basil variety characterized by compact growing habit and heavily cup-shaped leaves, were transplanted in two microcosm devices, each one equipped with 6 cylindrical pots (60 cm height × 20 cm diameter; 25 cm spaced out from each other) and set up as described in d’Aquino et al. [33]. In each cylindrical pot previously filled with commercial potting soil (60% blond peat, 20% brown peat, 20% pumice 3–6 mm, pH 6.5), three seedlings were jointly transplanted. Environmental conditions were 20–26 °C (night–day) with 60% relative humidity and 18–22 °C (night–day) in the epigeal and hypogeal chambers, respectively. Each microcosm was equipped with 6 square lamps, specifically designed and supplied by Becar S.r.l. (Beghelli group), carrying different LED arrays and enabling fine regulation of light spectra and PPFD. In the ‘microcosm white’ (W) only LEDs Luxeon SunPlus 20 Cool White (Lumileds, Schiphol, The Netherlands) and Oslon^®^ SSL 80 Cool White (Osram Opto Semiconductor, Regensburg, Germany) were activated, whereas in the ‘microcosm blue-red’ (BR) only LEDs, Royal Blue 445–455 nm and Deep Red 655–670 nm (Lumileds) and LEDs Oslon^®^ SSL 80 Deep Blue 451 nm and HyperRed 660 nm (Osram Opto Semiconductor) were activated. Figure 10 reports the spectral distributions of the two lighting setups in the region λ 350 ÷ 800 nm at 87 cm distance from the light source, i.e., at the seedlings level, determined using a spectroradiometer OL-770VIS (Gooch and Housego, Ilminster, UK) equipped with an Optopolymer integrating sphere.

The PPFD level was determined using a LI-190R Quantum Sensor and a LI-1500 Light Sensor Logger (LI-COR Biosciences, Lincoln, NE, USA) and it was 255 μmol m^−2^ s^−1^ at the seedling level in both the microcosms. The PPFD level under BR light was obtained from about 224 μmol m^−2^ s^−1^ from red light and from about 32 μmol m^−2^ s^−1^ from blue light (1:7). Photoperiodical conditions were 16/8 h light/dark. To prevent any external effect on the light supplied in the epigeal chambers, appropriate shadowing of the two microcosms was implemented. The plants were watered with 8.5 l water/pot, batching the total amount along the entire cultivation period according to the biomass growth, and fertilized twice with Fertiactyl GZ^®^ (Timac) (0.5% and ammonium sulphate 1 g l^−1^) during the growing cycle. To exploit the whole vegetative growth potential of plants, the aerial parts were harvested 58 days after transplant, when all the plants were at the beginning of the flowering stage, i.e., when the emission of inflorescence axis had started and before the anthesis had occurred, to prevent hijacking of leaf and stem resources by the reproductive organs.

### 4.2. Biometric Determinations

Plants (n = 3) in each pot were considered as one replicate. At the harvest stage (58 days after the transplant), the plant heights and the number of leaves per plant were recorded. Leaves, stems and inflorescence axes were then collected, and fresh and dry weights were determined. Samples from each pot were jointly collected. The analysis was performed on a total of 6 groups of plants per light treatment.

### 4.3. Chlorophyll Fluorescence Analysis

Photochemical parameters were measured on intact fully expanded leaves 28 days after transplant using a Mini version of the Imaging-PAM fluorimeter (Heinz Walz GmbH, Effeltrich, German), as described in d’Aquino et al. [33].

Photochemical parameters were measured on intact fully expanded leaves 28 days after transplant using a Mini version of the Imaging-PAM fluorimeter (Heinz Walz GmbH, Effeltrich, German), as described in d’Aquino et al. [33].

### 4.4. Ion Chromatography Analysis

Plant samples were allowed to dry in a ventilated environment at room temperature until a constant weight had been reached. Dried plant organs (stem and leaves) previously pooled from each pot were extensively grinded using a Retsch MM400 ball mill (Verder Scientific, Pedrengo, Italy). Each sample (0.5 g) was treated with 20 mL of ultrapure water in an ultrasonic bath twice for 30 min, to extract the soluble fraction; solutions were then filtered with pre-syringe filters with a porosity of 0.2 µm. One ml of each solution was then added to 100 μL of H_2_O_2_ and treated for 20 min in a 705 UV Digester (Metrohm, Origgio, Italy) to digest the organic fraction. Finally, volumes were corrected to 10 mL (dilution 1:10) with ultrapure water. The concentrations of major ions were determined by ion chromatography (IC) using a Dionex ICS1100 system (Thermo Fisher Scientific, Waltham, MA, USA). The detection of NO_3_^−^, PO_4_^3−^, SO_4_^2−^ and Cl^−^ was performed using an ASRS 300-4 mm suppressor with a current of 33 mA, a AS22 column working with a cell volume of 100 µL and a buffer solution of 3.5 mM of sodium carbonate/bicarbonate as eluent, at a flow rate of 1.20 mL/min. The detection of Na^+^, K^+^, NH_4_^+^, Mg^2+^ and Ca^2+^ was performed using a CERS 500-4 mm suppressor with a current of 15 mA, a CS12A column working with a cell volume of 25 µL and a 20 mM methane sulfonic acid solution as eluent, at a flow rate of 0.25 mL/min. Calibration curves were calculated using certified multistandard solutions. The detection of NH_4_^+^, NO_3_^−^, PO_4_^3−^, SO_4_^2−^, Ca^2+^, Mg^2+^, Na^+^ and Cl^−^ was performed on dried samples, as already reported [33]. The contents of N, P and S in relation to their corresponding inorganic soluble fractions were calculated using the molar mass of the elements from the concentrations of their ionic chemical forms (NO_3_^−^ + NH_4_^+^, PO_4_^3−^ and SO_4_^2−^, respectively).

### 4.5. Plant Extraction and Hyphenated Liquid Chromatography High-Resolution Mass Spectrometry (LC-ESI-Orbitrap-MS) Analysis

Intact apical and middle leaves were independently collected from plants randomly selected in the two microcosms. Dried plant leaves were ground using liquid nitrogen and 150 mg of the powder was homogenized with 2 mL of a solution of ethanol and water (1:1). Extracts were sonicated for 10 min and centrifuged at 3000 *g*. Supernatants were dried under nitrogen flow and then diluted with 2 mL of methanol. To remove chlorophyll, 1 mL of extract was subject to solid phase extraction using a Strata^®^ SCX 55 µm, 70 Å cartridge (Phenomenex, Torrance, CA, USA) preconditioned with methanol. Elution of samples was performed using 1 mL of methanol. The eluted samples were evaporated under nitrogen flow and dissolved in methanol/water (1 mg/mL) and 10 µL was injected in the LC-MS system. LC-MS analysis and molecule identification were carried out as detailed by d’Aquino et al. [33]. For the fragmentation studies, a data-dependent scan experiment was performed to select precursor ions corresponding to the most intense peaks in LC-MS analysis. Xcalibur software version 2.1 was used for instrument control, data acquisition and data analysis.

### 4.6. Data Analysis

Data from biometric determination, chlorophyll fluorescence measures and ion concentrations were analysed by one-way Anova using the SPSS 27 software package (www.ibm.com/software/analytics/spss 20 November 2022). For the metabolomics analyses, multivariate data analysis was carried out as described by Sarais et al. [73]. Raw LC-ESI/LTQOrbitrap/MS data were analysed by MZmine software (http://mzmine.sourceforge.net/ 20 November 2022). The resulting data matrices from untargeted (48 observations and 2500 variables) and pseudo-targeted (48 observations and 30 variables) analyses were processed using Umetrics SIMCA-P+ software 12.0 using PCA (Principal Component Analysis) for visualization and PLS-DA (Partial Least Square—Discriminant Analysis) for classification.

## 5. Conclusions

In this study, higher biomass yield was recorded under W light compared to BR light in fully expanded basil plants, possibly because the W light ensures a better reach of inner and lower parts in plants in which the epigeal part displays a complex aerial architecture, with many leaf layers and extensive lateral shooting. This finding should be taken into consideration in indoor basil farming targeted to the production of fully expanded plants, in which canopy complexity increases during the cultivation period, and it is also emphasized by lateral shooting that occurs after successive harvesting of leaves. Even if a definitive relationship between metabolic profiles and basil quality is yet to be deciphered, the results from metabolomics analysis confirmed that the light spectrum affects secondary metabolism in basil and provided novel information about the metabolic profile of fully expanded basil plants. Several potentially bioactive phenolic compounds were recorded at a higher level in plants grown under BR light and this finding strengthens the hypothesis that light modulation can provide farmers with a tool to drive the basil metabolic profile according to specific aims. The two tested lighting regimes also differently affected translocation rate and leaf accumulation of minerals that are related to basil final quality, particularly nitrate ions. Overall, our results suggest that W light can be preferred in the early stages of basil cultivation to enhance biomass production, while BR light can be supplied in the late cultivation period to decrease nitrate content and to increase the content of beneficial mineral nutrients and phenolic compounds in the leaves. Nevertheless, further investigations under microcosm conditions using additional wavelengths, different PPFD levels and different photoperiodic conditions are needed to assess new lighting approaches suitable to increase the yield and to drive secondary metabolism in basil.

## Figures and Tables

**Table 1:** Average concentrations (mg∙g^−1^) ± SE (n = 3 replicates) of the soluble fractions of the main nutrients plus Na^+^ and Cl^−^ ions in leaves and stems of basil plants grown under W and BR light. *p* values are shown; not significant and significant differences at *p* ≤ 0.01 or 0.001 are reported as ns, ** and ***, respectively.

		Ion Concentrations (mg g^−1^)												
		N	NH_4_^+^	NO_3_^−^	P	PO_4_^3−^	K^+^	Mg^++^	Ca^++^	S	SO_4_^2−^	Na^+^	Cl^−^	∑ions
Leaves	W	6.21 ± 0.04	1.17 ± 0.04	23.47 ± 0.04	2.071 ± 0.009	6.35 ± 0.03	49.58 ± 0.03	3.72 ± 0.03	3.49 ± 0.02	1.33 ± 0.00	4.00 ± 0.01	0.01 ± 0.01	12.01 ± 0.01	113.37 ± 0.25
	BR	5.95 ± 0.02	1.57 ± 0.02	20.96 ± 0.02	2.619 ± 0.004	8.03 ± 0.01	46.54 ± 0.02	4.34 ± 0.02	4.93 ± 0.03	1.49 ± 0.01	4.48 ± 0.02	0.01 ± 0.01	11.37 ± 0.02	112.30 ± 0.20
Stems	W	4.12 ± 0.05	0.87 ± 0.05	15.25 ± 0.02	1.451 ± 0.006	4.45 ± 0.02	39.28 ± 0.01	1.94 ± 0.02	2.17 ± 0.02	0.76 ± 0.00	2.29 ± 0.01	1.72 ± 0.02	6.94 ± 0.01	81.25 ± 0.24
	BR	3.01 ± 0.00	0.80 ± 0.00	10.58 ± 0.01	1.414 ± 0.007	4.33 ± 0.02	28.68 ± 0.01	1.45 ± 0.01	1.76 ± 0.01	0.88 ± 0.00	2.64 ± 0.01	0.92 ± 0.02	4.61 ± 0.02	61.09 ± 0.12
Leaves/Stems ratio	W	1.51	1.34	1.54	1.428	1.43	1.26	1.92	1.59	1.74	1.74	0.01	1.73	1.39
	BR	1.98	1.95	1.98	1.853	1.85	1.62	3	2.8	1.7	1.7	0.01	2.46	1.84
Significance		N	NH_4_^+^	NO_3_^−^	P	PO_4_^3−^	K^+^	Mg^++^	Ca^++^	S	SO_4_^2−^	Na^+^	Cl^−^	∑ions
Leaves		0.00 **	0.00 **	0.00 **	0.00 **	0.00 **	0.00 **	0.00 **	0.00 **	0.00 **	0.00 **	0.895 ns	0.00 **	0.006 **
Stems		0.00 **	0.107 **	0.00 **	0.002 **	0.002 **	0.00 **	0.00 **	0.00 **	0.00 **	0.00 **	0.00 **	0.00 **	0.002 **
Leaves/Stems ratio		0.00 **	0.001 ***	0.00 **	0.00 **	0.00 **	0.00 **	0.00 **	0.00 **	0.00 **	0.003 **	0.527 ^ns^	0.00 **	0.00 **

**Table 2:** Compounds identified by LC-ESI-Orbitrap-MS and MS/MS analyses in negative ion mode numbered in order of elution.

N.	R_T_	[M-H]^−^	Molecular Formula	Δ ppm	MS/MS	Identity
**1**	2.37	227.0611	C_7_H_13_O_8_	2.6	179	caffeic acid + formiate adduct
**2**	3.86	263.0043	C_8_H_7_O_10_	4.7	244	sugar
**3**	3.86	355.1237	C_13_H_23_O_11_	1.9	193	sugar
**4**	10.20	593.1497	C_27_H_29_O_15_	0.5	353/473	vicenin 2
**5**	12.47	197.0454	C_9_H_9_O_5_	3.09	153/129	syringic acid
**6**	12.56	463.0868	C_21_H_19_O_12_	0.5	301	quercetin 3-*O*-glucoside
**7**	12.81	300.9982	C_14_H_5_O_8_	1.01	283/257/233/227	ellagic acid
**8**	13.22	473.0718	C_22_H_17_O_12_			chicoric acid
**9**	13.80	353.0872	C_16_H_17_O_9_	1.4	-	cholorogenic acid
**10**	13.85	665.3887	C_36_H_57_O_11_		621/431	triterpene
**11**	13.96	311.1130	C_15_H_19_O_7_		183/267/293	1-*O*-β-d-glucopyranosyloxy-2-hydroxy-4-allylbenzene
**12**	14.39	475.0869	C_22_H_19_O_12_	−0.3	285	luteolin 7-*O*-glucuronide-6′-methyl ester
**13**	14.61	359.0760	C_18_H_15_O_8_	1.02	161	rosmarinic acid
**14**	15.09	738.3691	C_37_H_56_O_14_N	0.7	648/283	unknown
**15** **16**	15.64 15.80	459.0923 327.0868	C_22_H_19_O_11_ C_18_H_15_O_6_	0.35 1.5	269 185/199/283/309	apigenin 7-*O*-glucuronide methyl ester
**17**	15.93	193.0505	C_10_H_9_O_4_	5.04	161/134/178	ferulic acid
**18**	16.00	565.1550	C_26_H_29_O_14_	0.1	367/197	unknown
**19**	16.40	227.0708	C_14_H_11_O_3_	17		resveratrol
**20**	17.48	373.0922	C_19_H_17_O_8_	1.4	179/135	rosmarinic acid methyl ester
**21**	18.74	327.0868	C_18_H_15_O_6_	1.5	185/199/283/309	salvigenin
**22**	18.74	565.1343	C_29_H_25_O_12_	0.72	533/353	unknown
**23**	19.14	311.1680	C_13_ H_27_ O_8_	0.82	183	unknown
**24**	19.28	357.0971	C_19_H_17_O_7_	0.6	339/313/289	gardenin b
**25**	20.00	501.1026	C_24_H_21_O_12_		339/161	di-feruloyl-tartaric acid
**26**	20.88	289.0687	C_15_H_13_O_6_	−6.9		catechin
**27**	28.30	291.1989	C_15_H_31_O_3_S	1.6	198	sulfurous acid
**28**	29.20	309.1734	C_14_H_29_O_5_S	1.7	96	ethanol, 2-(dodecyloxy), 1-(hydrogen sulfate)
**29**	30.91	455.3515	C_30_H_47_O_3_	−0.15	283/193	oleanolic acid
**30**	37.60	455.3517	C_30_H_47_O_3_	−0.15	283/199	ursolic acid
